# Muscle synergy asymmetry between the affected and unaffected limbs during the stance phase in unilateral flatfoot

**DOI:** 10.1186/s12984-026-01995-8

**Published:** 2026-05-02

**Authors:** Jianqi Pan, Zixiang Gao, Zhanyi Zhou, Dongxu Wang, Fengping Li, Diwei Chen, Zsolt Radak, Yaodong Gu

**Affiliations:** 1 Department of Rehabilitation Medicine, Ningbo No. 2 Hospital, Zhejiang Ningbo, China; 2 Faculty of Sports Science, Ningbo University, No. 818, Fenghua Road, Jiangbei District, Zhejiang 315211 Ningbo, China; 3 Faculty of Kinesiology, University of Calgary, Calgary, Canada; 4 Research Institute of Sport Science, Hungarian University of Sports Science, Budapest, Hungary

**Keywords:** Flatfoot, Gait, Muscle synergy, Vector coding, Coupling angle

## Abstract

**Background:**

Musculoskeletal conditions can disrupt neuromuscular coordination during gait, leading to asymmetry and altered muscle synergy patterns. Understanding how the nervous system reorganizes synergies in individuals with unilateral flatfoot (UF) is essential for improving clinical assessment and rehabilitation strategies.

**Methods:**

Surface electromyography (sEMG) of major lower-limb muscles during the stance phase was collected from 24 individuals with UF. Synergists were extracted by non-negative matrix factorization (NMF) and analyzed with vector coding (VC) to examine bilateral phase relationships. Independent t-tests compared muscle weights within synergies, paired t-tests compared temporal waveforms between sides, and chi-square tests examined differences in dominant quadrant distribution.

**Results:**

Both affected (AFF) and unaffected (UNAFF) sides exhibited two synergies. The tibialis anterior (TA) (Syn1: AFF = 0.47 ± 0.40, UNAFF = 0.46 ± 0.38; Syn2: AFF = 0.36 ± 0.38, UNAFF = 0.46 ± 0.39) and medial gastrocnemius (MG) (Syn1: AFF = 0.41 ± 0.40, UNAFF = 0.49 ± 0.43; Syn2: AFF = 0.52 ± 0.41, UNAFF = 0.45 ± 0.41) showed significantly higher weights than the mean value within each respective synergy (*p* < 0.001). Both sides mainly exhibited an In-phase pattern, with Syn1 more prevalent on the UNAFF side (*p* < 0.05) and Syn2 on the AFF side (*p* < 0.05).

**Conclusion:**

The combined NMF-VC analysis effectively captured the structural and temporal features of muscle synergies. UF individuals exhibited MG compensation on the AFF side, suggesting that nervous system may maintaining stability through strengthening synergy patterns dominated by major muscles. This study revealed the neuromuscular reorganization in UF gait and provided a quantitative method for synergy-based rehabilitation assessment.

## Background

Flatfoot refers to a foot deformity where the inner longitudinal arch is completely or partially lost [1]. This abnormal structural will alter the biomechanics of the foot, thereby compromising the postural stability [2] and increasing the risk of lower limb injuries [3]. Compared with normal feet, individuals with flatfoot exhibit characteristics such as a large peak of forefoot plantar flexion and a decreased peak of foot adduction during gait [4]. Long-term abnormal foot postures may lead to pain and injury [4, 5], which in turn affects the pattern of lower limb muscles. Prior work [6] also reported significant differences in muscle activation between flatfoot and normal foot conditions. Previous studies have mainly focused on the overall gait characteristics of individuals with bilateral flatfoot, whereas research on unilateral flatfoot (UF) remains limited. UF individuals show structural and functional differences between the affected (AFF) and unaffected (UNAFF) sides during gait [7]. It has been reported that the occurrence of UF is not dependent on spinal asymmetry but is often accompanied by varying degrees of pelvic tilt and spinal postural deviation, which may be related to altered lower-limb load distribution and muscle control mechanisms [8]. Comparing the AFF and UNAFF within the same individual can effectively reduce inter-individual variability, and a similar research design has been applied to unilateral lower-limb pathological models such as unilateral knee osteoarthritis [9].

The simultaneous activation of multiple muscles during gait is commonly referred to as muscle synergy [10]. The muscle synergy theory suggests that the nervous system controls complex multi-joint movements by activating a limited number of synergy modules, reflecting a neural strategy for simplifying motor control [11]. The neural control of human gait may follow a modular control mechanism [12]. However, muscle synergies can also exhibit a certain degree of plasticity, which can make adaptive adjustments to pathological factors and exhibit individual differences [13]. UF individuals may represent a typical example of this mild synergistic change. Muscle synergy analysis is currently regarded as the most effective method for assessing sensorimotor deficits [14], and it can quantitatively characterize the neural control features of limb movements [11]. The gait differences of UF individuals can be accurately expressed through this method. Muscle synergy activity is typically recorded by electromyography (EMG), while surface electromyography (sEMG) can also reveal the synchronous activation patterns within synergistic muscle groups [15, 16]. Among various algorithms, non-negative matrix factorization (NMF) has been recognized as one of the most effective methods for identifying muscle synergies in walking tasks [17]. It decomposes the sEMG signals from multiple muscles into several synergy modules, thereby identifying their constituent weights and activation temporal features. Therefore, this study employs NMF to characterize the differences in muscle synergy between the AFF and UNAFF limbs of UF individuals during the stance phase.

Muscle synergy analysis provides an important method for understanding the modular control strategies within the nervous system, enabling effective identification of fundamental units of motor function (synergy patterns) and their activation characteristics [11]. To fully comprehend the motor control process, it is not only necessary to identify the synergistic modules themselves, but also to elucidate the mechanisms of interaction between them. NMF analysis is difficult to reveal the real-time interactions among different synergy patterns throughout the stance phase [18]. Therefore, it has limitations in quantifying the dynamic coordination relationships among synergy modules. To address this limitation, this study introduces the vector coding (VC) method as a complementary analytical approach to achieve a higher-level analysis of coordination dynamics. VC is a nonlinear data analysis technique that can be used to quantify intersegmental coordination and coordination variability [19]. Previous studies [20–22] mostly applied VC to joint angle or segment angle data, but as a mathematical approach, VC itself is independent of data type. The VC method can measure the continuous dynamic interaction between two time series signals by determining the vector direction between two adjacent data points [23]. The modified VC method can express the relationship between two seemingly complex activation curves using a single and clear indicator, the coupling angle (CA) [24]. By calculating the CA, the relative phase relationships (in-phase, anti-phase) between synergistic activation patterns can be precisely quantified, thereby describing their coordination patterns and temporal distribution [19]. This method provides a reliable quantitative tool for this study to directly compare the temporal features of neural control at the synergy level between bilateral lower limbs. In this study, the synergy modules extracted by NMF were used as inputs to calculate CA, which was then mapped to the range of 0° ~ 360° to intuitively reveal the dominant relationships between synergy modules.

This study aims to compare the phase relationship between the synergistic activation patterns of lower-limb core muscles of UF individuals during the stance phase of gait using the coupling analysis method of NMF and modified VC. The goal is to quantify and reveal, from a neuromuscular coordination perspective, the bilateral differences in motor control timing and potential compensatory mechanisms. It is hypothesized that the neural control pattern of the UNAFF differs from that of the AFF, with the UNAFF likely enhancing early-phase synergy activation to support body loading and propulsion, while the AFF tends to employ compensatory control at later phases to maintain gait stability.

## Methods

**Fig. 1:**
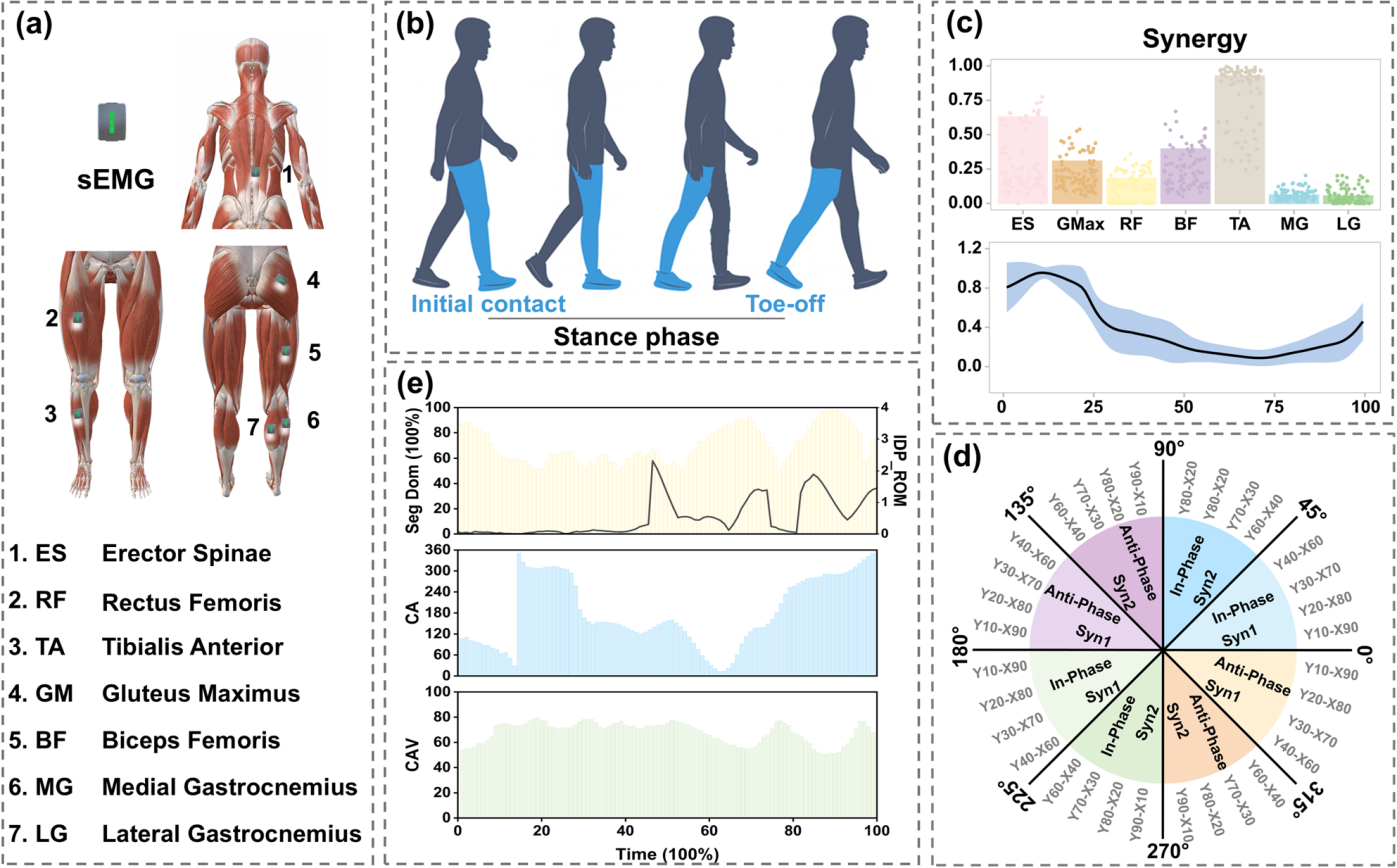
Schematic overview of the analysis procedure. (**a**) Placement of surface electromyography (sEMG) electrodes (unilateral). (**b**) Illustration of the stance phase for one limb during gait. (**c**) Extraction of muscle synergies based on non-negative matrix factorization (NMF). The upper panel shows the weight distribution of each muscle within the synergy, and the lower panel presents the temporal activation profiles of the synergies throughout the normalized stance phase (0 ~ 100%). (**d**) Classification of coordination patterns based on coupling angle (CA). Rotations in the same direction are defined as In-phase, and those in opposite directions as Anti-phase. Each quadrant represents a coordination pattern, with colors indicating different dominance relationships. The first synergy (Syn1) is defined as the X-axis variable (X), and the second synergy (Syn2) as the Y-axis variable (Y). Blue and green denote In-phase, while orange and purple denote Anti-phase; lighter colors indicate Syn1 dominance, and darker colors indicate Syn2 dominance. (**e**) Illustration of segment dominance percentage (Seg Dom), coupling angle (CA), coupling angle variability (CAV), and inter-data-point range of motion (IDP-ROM, shown as black lines) across the normalized stance phase (0 ~ 100%)

Biomechanical data of the lower limbs were collected from 24 UF individuals during normal walking. The data were then processed to analyze bilateral muscle activation patterns during the stance phase. Subsequently, the CA was calculated to determine the corresponding coordination pattern and dominant synergy component (Fig. 1).

### Participants

A total of 24 individuals (16 males and 8 females) with unilateral flatfoot were recruited for this study (Table 1). None of the participants had a history of lower-limb disorders within the past six months or severe sports injuries within the past year. Foot morphology was assessed using an Easy-Foot-Scan system (Easy-Foot-Scan, OrthoBaltic, Kaunas, Lithuania). The arch index (AI) was calculated by dividing the midfoot contact area by the total plantar area (excluding the toes), and an AI ≥ 0.26 was considered indicative of flatfoot [25]. All participants voluntarily signed written informed consent forms prior to participation, and the study protocol was approved by the Ethics Committee of Ningbo University (TY2025046).

**Table 1:** Basic information of participants

Age (year)	Height (cm)	Weight (kg)	Arch height (mm)		Arch index	
			AFF	UNAFF	AFF	UNAFF
23.67 ± 1.75	168.92 ± 9.99	68.18 ± 17.24	9.20 ± 1.52	14.42 ± 1.74	0.27 ± 0.01^a^	0.24 ± 0.01^a^

^a^ denotes a significant difference between sides based on a paired t-test (*p* < 0.001, Cohen’s *d* = − 2.556)

### Procedure

A total of 38 reflective markers were placed on each participant’s body according to the Gait-2392 model (excluding head markers). Kinematic and kinetic data during the entire walking process were recorded using a Vicon infrared motion capture system composed of 10 cameras (200 Hz, Oxford Metrics Ltd, Oxford, UK), synchronized with Kistler force plates (1000 Hz, Kistler Instrumente AG, Winterthur, Switzerland). After participants became familiar with the laboratory environment, they were instructed to walk naturally along a 10 m walkway. A trial was considered valid when the UNAFF and AFF sides each made full contact with different force plates, and complete bilateral stance-phase data were successfully captured. sEMG signals were recorded using a 16-channel Delsys system (1000 Hz, Delsys Inc., Natick, MA, USA) and synchronized with the Vicon system via an A/D converter (Vicon Motion Systems, Oxford, UK). Following the SENIAM recommendations [26], sEMG data were collected from 14 muscles (seven per side) (Fig. 1a; Table 2): erector spinae (ES), gluteus maximus (Gmax), rectus femoris (RF), biceps femoris (BF), tibialis anterior (TA), medial gastrocnemius (MG) and lateral gastrocnemius (LG).

**Table 2:** Detailed anatomical landmarks for sEMG electrode placement

Muscle	Sensor locations
ES	At two finger widths lateral to the spinous process of the L1 vertebra
Gmax	At the midpoint of the line between the sacrum and the greater trochanter
RF	At the midpoint of the line between the ASIS and the superior border of the patella
BF	At the midpoint of the line between the ischial tuberosity and the lateral epicondyle of the femur
TA	At 1/3 of the distance on the line between the fibular head and the medial malleolus
MG	On the most prominent part of the medial gastrocnemius muscle belly
LG	At 1/3 of the distance on the line between the fibular head and the calcaneus

ES, Gmax, RF, BF, TA, MG, and LG denote erector spinae, gluteus maximus, rectus femoris, biceps femoris, tibialis anterior, medial gastrocnemius, and lateral gastrocnemius, respectively. All electrodes were placed on the muscle belly and oriented parallel to the direction of the muscle fibers

### Data processing

Raw sEMG signals from both sides during the stance phase were preprocessed using MATLAB R2024a (The MathWorks, Natick, MA, USA). Baseline correction was performed by applying a fourth-order Butterworth high-pass filter at 20 Hz to remove low-frequency drift. Full-wave rectification was then performed, followed by a fourth-order Butterworth low-pass filter with a cutoff frequency of 10 Hz to extract the linear envelope. This filtering protocol was based on previous research regarding lower-limb muscle synergy during gait [16]. To minimize inter-individual variability in muscle activation, all sEMG signals were amplitude-normalized to the peak activation amplitude of each muscle within the stance phase [27]. Meanwhile, the stance phase of each subject was time-normalized to 101 data points (0 ~ 100% stance phase) to facilitate subsequent calculations.

### NMF and VC

To extract muscle synergy modules, the normalized sEMG signals were decomposed using NMF [17]:

1 $$\:A\left(t\right)\approx\:\sum\:_{k=1}^{{N}_{syn}}\:{B}_{k}\left(t\right){W}_{k}$$

where $\:{N}_{syn}$ is the number of extracted synergy modules, $\:{W}_{k}$ represents the weight vector of the $\:k$-th synergy, and $\:{B}_{k}\left(t\right)$ denotes its temporal activation coefficient. For each participant and each limb, 1 ~ 7 synergy modules were successively extracted from the unilateral data to determine the optimal number of synergies ($\:{N}_{syn}$). $\:{N}_{syn}$ was defined as the smallest number of synergies required to achieve approximately 95% of the EMG reconstruction coefficient of determination ($\:{R}^{2}$) [28], calculated as follows [29]:

2 $$\:{R}^{2}=1-\frac{SSE}{SST}$$

3 $$\:\left\{\begin{array}{c}SST=\sum\:_{i,j}\:{\left({D}_{ij}-\mu\:{D}_{i}\right)}^{2}\\\:SSE=\sum\:_{i,j}\:{\left({D}_{ij}-[WC{]}_{ij}\right)}^{2}\end{array}\right.$$

where $\:{D}_{ij}$ is the sEMG value of the $\:i$ muscle at the $\:j$ time point, $\:\mu\:{D}_{i}$ is the mean value of that muscle, $\:SST$ represents the total sum of squares, and $\:SSE$ represents the residual sum of squares from reconstruction. To avoid local minima, the NMF algorithm was executed for 20 independent runs with random initializations of $\:W$ and $\:B\left(t\right)$. Within each run, the matrices were iteratively updated to optimize the synergy modules. A single run was terminated if the improvement in $\:{R}^{2}$ remained below 0.001% over 20 consecutive iterations. After the completion of all 20 independent runs, the solution with the highest $\:{R}^{2}$ was selected for further analysis [29]. An $\:{N}_{syn}$ value of two was the most frequent optimal choice across all participants. Subsequent retrospective verification confirmed that $\:{N}_{syn}$ = 2 met the $\:{R}^{2}$ ≥ 95% threshold for all individual limbs. Therefore, it was adopted as the representative synergy number to ensure consistency in group-level analysis [30]. To facilitate group-level comparison, the extracted individual synergy weight vectors ($\:W$) were clustered using the K-means algorithm (*k* = 2) with squared Euclidean distance to align functionally similar modules across participants [31, 32]. Synergies assigned to the same cluster were categorized as the same functional module. Group-level synergy weights were then calculated as the mean of these aligned individual weights, with error bars representing the inter-subject standard deviation (SD).

Mathematically, while NMF identifies distinct temporal activation profiles of motor modules and represents them as separate activation curves, it primarily reflects the activation magnitude of individual synergies and lacks a direct metric to quantify their instantaneous coordination or coupling at a given time point. However, successful gait relies on precise coordination and smooth transitions between these functional modules [12]. The VC method addresses this limitation by calculating the vector direction of changes in the activation coefficients of two synergy modules across adjacent time points, thereby quantifying their real-time coordination dynamics and neural coupling characteristics that cannot be captured by NMF alone. Therefore, to further examine the dominance differences between sides within the synergy patterns, the CA and coupling angle variability (CAV) were calculated using the VC method. The CA was defined as the vector direction formed by the changes in the temporal activation coefficients of the two synergies between adjacent time points during the stance phase, reflecting the relative phase relationship between the two synergies [24]. As a mathematical approach, VC is independent of data type and has been previously applied to the analysis of coordination between upper and lower limbs as well as between contralateral limbs [33]. Since two synergies were extracted from both sides, the temporal activation coefficient of the first synergy (Syn1) was defined as the X-axis variable (X), and that of the second synergy (Syn2) was defined as the Y-axis variable (Y) to facilitate computation and interpretation. The calculation formula is as follows [19]:

4 $$\:{\gamma\:}_{i}=\mathrm{a}\mathrm{t}\mathrm{a}\mathrm{n}2\left({\Delta\:}{\theta\:}_{Xi},{\Delta\:}{\theta\:}_{Yi}\right)\cdot\:\frac{180}{\pi\:}$$

5 $$\:\left\{\begin{array}{c}\varDelta\:{\theta\:}_{Xi}={\theta\:}_{X(i+1)}-{\theta\:}_{Xi}\\\:\varDelta\:{\theta\:}_{Yi}={\theta\:}_{Y(i+1)}-{\theta\:}_{Yi}\end{array}\right.$$

Specifically, a CA ($\:{\gamma\:}_{i}$) oriented closer to the X-axis indicates that the change in activation is dominated by Syn1, while proximity to the Y-axis signifies dominance by Syn2. These relationships are quantified as the segment dominance percentage (Seg Dom), which represents the proportion of a gait phase during which one synergy module exerts a greater influence on the coordination pattern than the other (Fig. 1e). The CA ($\:{\gamma\:}_{i}$) was further normalized to a range of 0° ~ 360°:

6 $$\:{\gamma\:}_{i}=\left\{\begin{array}{c}{\gamma\:}_{i}+360,\hspace{0.25em}\hspace{0.25em}\hspace{0.25em}\hspace{0.25em}{\gamma\:}_{i}<0\\\:{\gamma\:}_{i},\hspace{0.25em}\hspace{0.25em}\hspace{0.25em}\hspace{0.25em}{\gamma\:}_{i}\ge\:0\hspace{0.25em}\hspace{0.25em}\hspace{0.25em}\hspace{0.25em}\end{array}\right.$$

the mean horizontal and vertical components ($\:\stackrel{-}{{X}_{i}}$, $\:\stackrel{-}{{Y}_{i}}$) were calculated across all trials, and the mean coupling angle (MCA) was derived from $\:\stackrel{-}{{X}_{i}}$ and $\:\stackrel{-}{{Y}_{i}}$:

7 $$\:\left\{\begin{array}{c}\stackrel{-}{{X}_{i}}=\frac{1}{n}\sum\:_{j=1}^{n}\:\mathrm{cos}{\gamma\:}_{ij}\\\:\stackrel{-}{{Y}_{i}}=\frac{1}{n}\sum\:_{j=1}^{n}\:sin{\gamma\:}_{ij}\end{array}\right.$$

8 $$\:\stackrel{-}{{\gamma\:}_{i}}=\mathrm{a}\mathrm{t}\mathrm{a}\mathrm{n}2(\stackrel{-}{{Y}_{i}},\stackrel{-}{{X}_{i}})\cdot\:\frac{180}{\pi\:}$$

similarly normalized to a range of 0° ~ 360°, the length of MCA was calculated using the following formula:

9 $$\:\stackrel{-}{{r}_{i}}=\sqrt{{\stackrel{-}{{X}_{i}}}^{2}+{\stackrel{-}{{Y}_{i}}}^{2}}$$

the CAV was further calculated:

10 $$\:CA{V}_{i}=\sqrt{2\cdot\:(1-\stackrel{-}{{r}_{i}})}\cdot\:\frac{180}{\pi\:}$$

the results were mapped onto four coordination patterns (In-phase, Anti-phase; Proximal dominance, Distal dominance). The CA mapping method was used to quantify the distribution within each dominant quadrant, thereby identifying whether Syn1 or Syn2 was dominant during different gait phases (Fig. 1d) [21]. In addition, the inter-data-point range of motion (IDP-ROM) for the dominant segment was quantified and superimposed onto the coupling angle mapping and segment dominance analysis (Fig. 1e). IDP-ROM serves as a metric to quantify the intensity of synergy activation changes, reflecting the modulation rate of neural drive during gait phase transitions [19]. Large IDP-ROM values typically correspond to a vigorous reorganization of synergy patterns, indicating that the nervous system is rapidly adjusting muscle activation to satisfy walking demands.

All analyses were performed using R v4.5.0 (R Foundation for Statistical Computing, Vienna, Austria).

### Statistical analysis

To compare differences between sides, statistical analyses were performed on both NMF and CA mapping results. Using SPSS 27.0.1 (IBM Corp, Armonk, NY, USA), a paired-sample t-test was utilized to compare the arch index between limbs to confirm bilateral structural asymmetry. Pearson correlation analysis was conducted between the synergy weights of the AFF and UNAFF sides after K-means alignment to verify the structural consistency of corresponding synergies. Independent-sample t-tests were further applied to compare individual muscle weights within each synergy against the intra-synergy mean weight to identify dominant contributors. For these dominant muscles, paired-sample t-tests were further used to compare their weights between the AFF and UNAFF sides. For the CA mapping results, a chi-square test of independence was used to examine overall distribution differences of dominant quadrants between sides. When a significant effect was found, Bonferroni-adjusted post hoc comparisons were performed to identify specific quadrant differences. In addition, paired-sample t-tests were performed using SPM1d in MATLAB specifically on the synergy temporal activation coefficients to evaluate temporal differences between the two sides. The significance level was set at 0.05 for all analyses.

## Results

### NMF analysis results

**Fig. 2:**
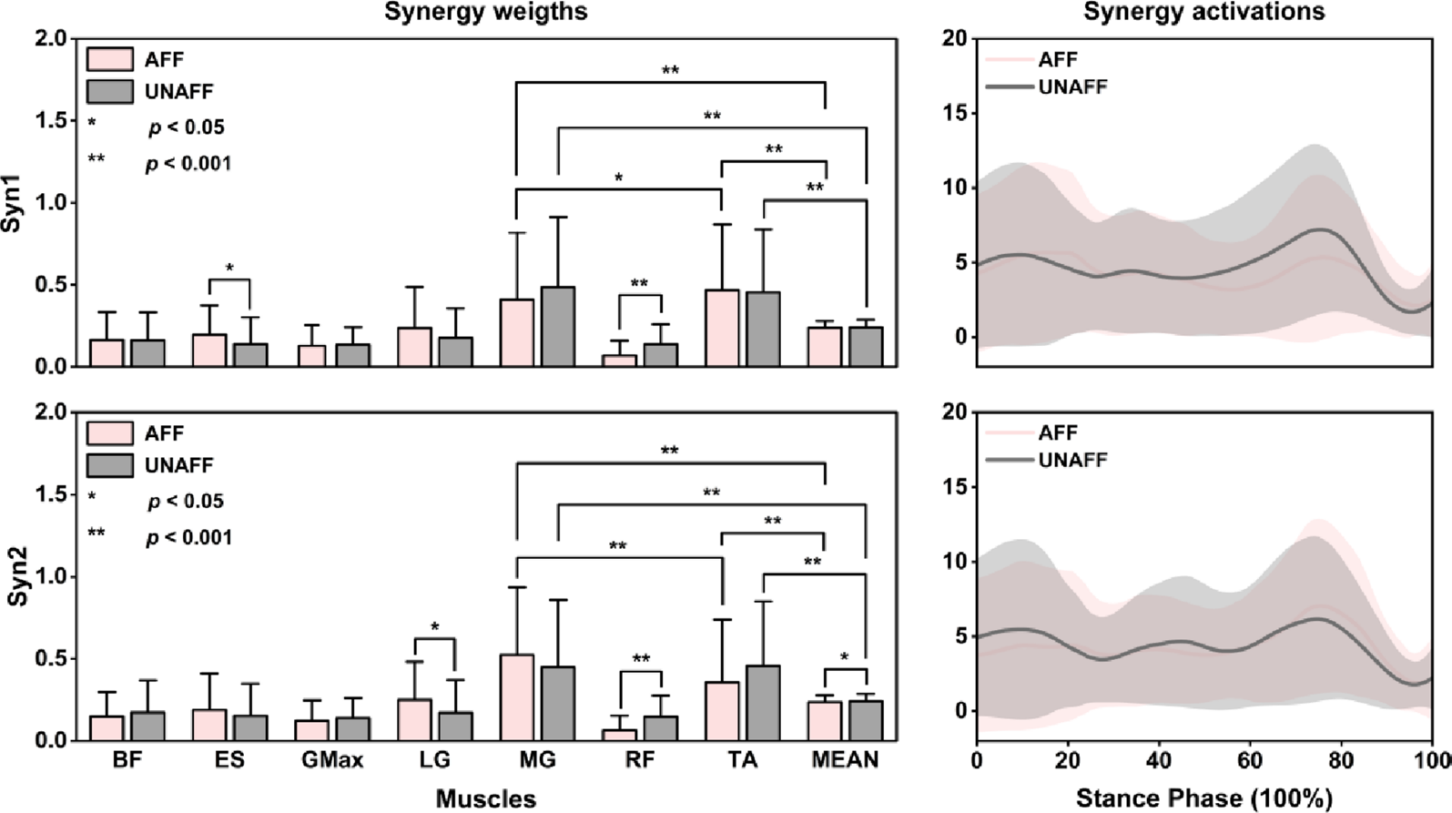
Weights and activation patterns of two muscle synergies (Syn1, Syn2) extracted using non-negative matrix factorization (NMF). The left panel shows comparisons of synergy weights between the affected (AFF) and unaffected (UNAFF) sides, as well as the comparison between individual muscle weights and the mean weight (MEAN) within each synergy; ES, Gmax, RF, BF, TA, MG, and LG represent erector spinae, gluteus maximus, rectus femoris, biceps femoris, tibialis anterior, medial gastrocnemius, and lateral gastrocnemius, respectively. The right panel illustrates the temporal activation curves of the synergies during the stance phase

Two synergies (Syn1, Syn2) were extracted from the AFF and UNAFF sides respectively through NMF. The consistency of the synergies was confirmed by Pearson correlation (Syn1: *r* = 0.94, *p* < 0.05; Syn2: *r* = 0.89, *p* < 0.05) and then compared (Fig. 2). The results showed that within both Syn1 and Syn2, the weights of MG and TA on both sides were significantly higher than the intra-synergy mean weight (*p* < 0.001). On the AFF side, Syn1 was mainly dominated by TA (*p* < 0.05), while Syn2 was dominated by MG (*p* < 0.001); the UNAFF side showed a similar dominance pattern, with no significant difference. Further comparison of muscle weights between sides revealed that the AFF side exhibited significantly higher ES weight in Syn1 and higher LG weight in Syn2 than the UNAFF side (*p* < 0.05), whereas the UNAFF side showed significantly higher RF weights in both synergies (*p* < 0.001). The temporal activation curves of both sides presented increased activation between 70% and 80% of the stance phase, with no significant difference between AFF and UNAFF.

### VC calculation results

**Fig. 3:**
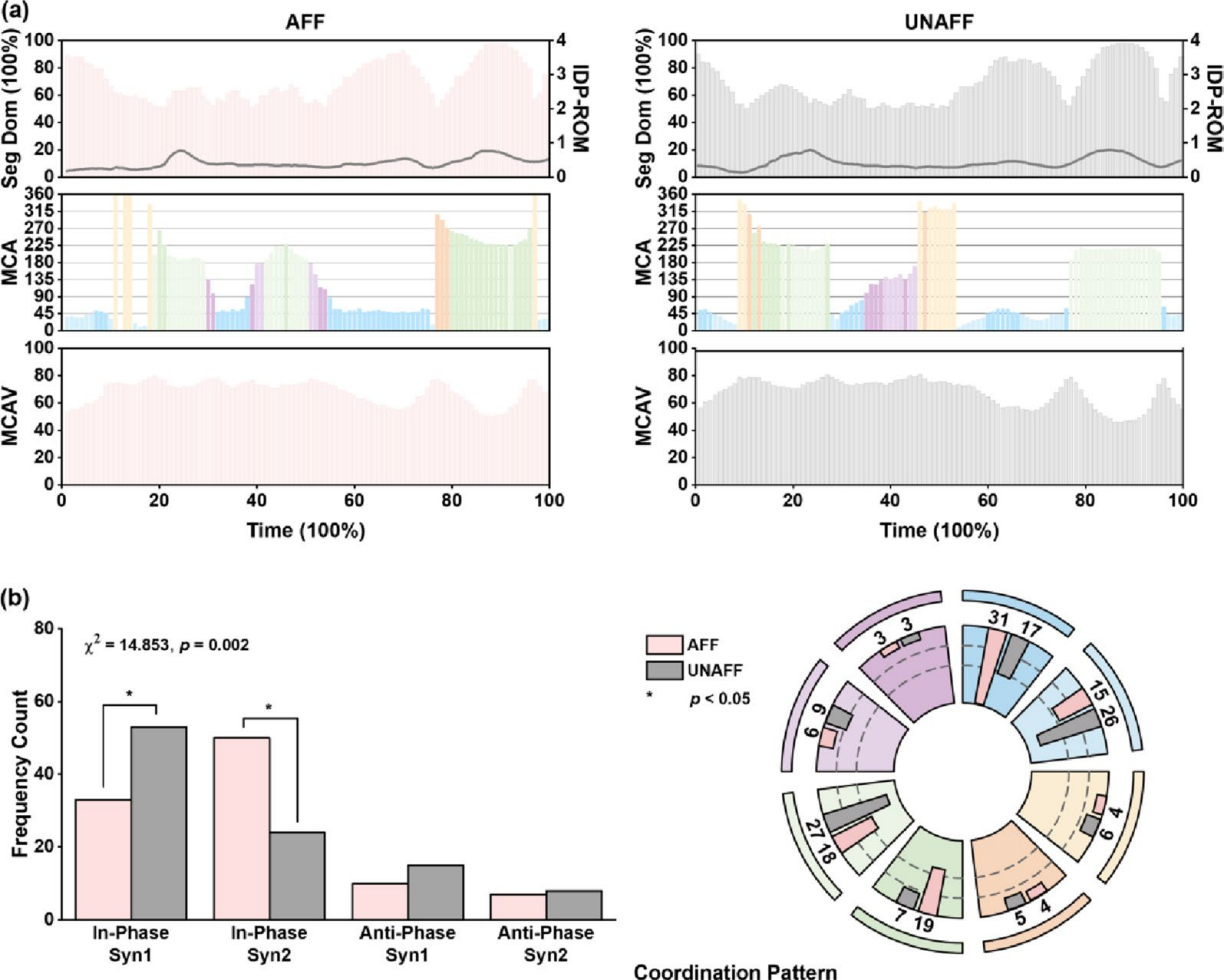
Coordination pattern analysis of AFF and UNAFF. (**a**) Temporal distributions of segment dominance percentage (Seg Dom), mean coupling angle (MCA), coupling angle variability (MCAV), and inter-data-point range of motion (IDP-ROM) during the normalized stance phase (0 ~ 100%). The color coding of MCA corresponds to the dominant color regions shown in Fig. 1d. (**b**) Frequency distribution of coordination patterns. The bar chart on the left illustrates frequency differences among coordination patterns, while the polar plot on the right displays their distribution across phase quadrants, with background colors corresponding to the dominant regions shown in Fig. 1d

The phase relationships between synergy components on the AFF and UNAFF sides during the stance phase are shown in Fig. 3a. On the AFF side, Seg Dom exceeded 80% with corresponding MCAV values below 60% during 0 ~ 6% (In-phase, Syn1), 63 ~ 72% (In-phase, Syn2), and 84 ~ 96% (In-phase, Syn2) of the stance phase, indicating stable coordination control. The AFF side exhibited higher IDP-ROM values (> 0.5) during 22 ~ 27%, 69 ~ 71%, and 83 ~ 94% of the stance phase, while remaining low in other intervals. On the UNAFF side, Seg Dom exceeded 80% with MCAV values below 60% during 0 ~ 3% (In-phase, Syn2), 62 ~ 71% (In-phase, Syn1), and 81 ~ 93% (In-phase, Syn1), also indicating stable coordination control. The UNAFF side showed higher IDP-ROM values (> 0.5) during 18 ~ 28% and 79 ~ 91% of the stance phase, with low values in other periods.

Statistical analysis of the frequency distribution under different coordination patterns (Fig. 3b, left) showed that both AFF and UNAFF were dominated by the In-phase pattern (AFF: 83, UNAFF: 77). A chi-square test revealed a significant overall difference between sides (χ^2^ = 9.088, *p* = 0.028). Post hoc Bonferroni analysis indicated that the difference primarily arose from the In-phase regions (first and third quadrants). The frequency count of Syn1-dominant phases was significantly higher for the UNAFF side than for the AFF side (*p* < 0.05), whereas the frequency count of Syn2-dominant phases was significantly higher for the AFF side than for the UNAFF side (*p* < 0.05). No significant differences were found in the remaining quadrants. The polar distribution (Fig. 3b, right) further characterizes these differences. Data points for the UNAFF side tended to cluster near the X-axis, exhibiting a Syn1-dominant In-phase pattern. In contrast, data points for the AFF side showed a noticeable shift toward the Y-axis, reflecting a Syn2-dominant In-phase characteristic. The frequency counts for anti-phase coordination were relatively low in both limbs (AFF: 17, UNAFF: 23).

## Discussion

This study, based on gait biomechanics data from individuals with UF, extracted bilateral muscle synergies using NMF and employed CA mapping to compare differences in muscle synergy and coordination patterns between the AFF and UNAFF sides during the stance phase. The results indicated that TA primarily dominated Syn1 and MG dominated Syn2 on the AFF side. The CA mapping analysis further revealed that the In-phase coordination pattern accounted for the largest proportion on both sides. Among them, the AFF side mainly exhibited the In-phase patterns dominated by Syn2, while the UNAFF side mainly showed the same patterns dominated by Syn1.

The primary mechanisms of flatfoot include traumatic joint instability, rupture of the posterior tibial tendon, and degenerative collapse of the first metatarsal-cuneiform joint [34]. A subsequent study [35] has found through imaging that adult primary flatfoot does not present with obvious lower limb rotational deformity. It’s the functional abnormalities may mainly result from compensatory neuromuscular regulation rather than bony structural deformity. This study is based on sEMG data and used NMF combined with VC to further explain the lower limb differences of UF at the neuromuscular level. The NMF results show that both AFF and UNAFF synergies were dominated by MG and TA, and both sides retain a similar neural synergistic control structure during gait. This finding aligns with a previous study [36] using conventional EMG, which reported that, despite variations in activation timing across populations, the TA and the gastrocnemius form the principal dorsiflexion-plantarflexion synergy during the stance phase. These results further confirm the simplified control strategy of human gait neural system, in which complex movement is achieved through the activation of a limited number of fixed muscle synergies [12]. Compared with the stable muscle synergy observed in the UNAFF side, the AFF side exhibited a clear differentiation of synergy modules, with Syn1 dominated by TA and Syn2 dominated by MG. During the stance phase, AFF may have adopted a phased muscle dominance pattern, achieving functional stability through alternating use of the anterior and posterior calf muscles. In healthy individuals, identified muscle synergies generally exhibit similar structure [31]. Under abnormal conditions, the synergy structure may undergo adaptive reorganization, forming different muscle patterns while still achieving similar motor functional outcomes [14]. The differentiated synergy pattern observed in the AFF side of this study can be interpreted as an indication of such neural control plasticity. A previous study [37] also demonstrated that human gait is generally maintained by relatively stable basic synergy modules, and that the nervous system can adjust its output through temporal overlap or reweighting under structural limitations.

Neural or musculoskeletal injuries can disrupt the consistency of the activation of synergy activation [38, 39]. This study found that the UNAFF side exhibited relatively stable coordination with well-defined temporal organization during the stance phase, whereas the AFF side showed certain phase fluctuations and control instability, possibly reflecting delayed or impaired synergy switching. The increased CAV in the affected limb likely reflects a decline in control stability caused by the loss of passive structural support. This fluctuation in coordination patterns is consistent with the searching behavior of the nervous system as it attempts to maintain stability under mechanical disadvantage [40]. A previous study [41] discovered that the affected side tends to activate multiple overlapping synergy modules to maintain functional stability, but this might also increase the burden of neural regulation. Similar compensatory strategies have been observed in the gait of individuals with stroke or neurological disorders [38]. Although the overall coordination pattern of the AFF side appeared more complex, both sides maintained low-variability In-phase control during critical stance phase, suggesting that the nervous system can preserve movement stability by reinforcing synergy coupling [42]. This was also found in previous studies on the coordinated activation of the upper limbs [43], although there were certain differences between the two sides, the activation coefficients still maintained a certain stability on both sides. In the counting results, both sides showed a dominant phase-locked (In-phase) coordination pattern during the stance phase of gait, and Syn1 and Syn2 showed overall synchronous activation. This may be attributed to the tendency of the nervous system to adopt a synchronized synergy control strategy during the stance phase [36], enabling the anterior and posterior muscle groups to jointly contribute to load bearing and propulsion under a unified timing pattern. However, differences in dominant synergies were observed between the two sides. The UNAFF side has a higher dominance of Syn1, while the AFF side has a higher dominance of Syn2. This dominance difference may stem from lower-limb asymmetry caused by UF. This has also been reported in a previous lower-limb study [44], where abnormal alignment was found to induce differences in muscle activation. The bilateral differences in dominant synergy distribution reveal neural adaptation mechanisms in individuals with flatfoot and can be further interpreted in relation to the functional roles of the synergy modules. On the UNAFF side, the weights of TA and MG remain relatively balanced, allowing Syn1 to facilitate shock absorption and eccentric control during load acceptance. In contrast, the distinct internal weighting observed in the AFF side reflects a reorganization of coordination strategies. Rather than a simple increase in muscle activation, the central nervous system reconfigures the relative contributions of muscles within each module to manage the loss of passive stability caused by medial arch collapse. This process is effectively characterized by IDP-ROM, which quantifies the magnitude of coordination change and represents the reconfiguration rate of neural drive during gait phase transitions. High IDP-ROM values during critical phases indicate a more intense reorganization of muscle synergies to compensate for the mechanical disadvantage of the foot. By shifting the internal balance toward a more polarized distribution, the nervous system potentially prioritizes modules that provide dynamic active support and enhance ankle joint stiffness. These findings underscore that the adaptation to structural deficits is achieved through the recalibration of coordination dynamics, where the internal organization of muscle weights is adjusted to maintain gait stability.

When gait stability is achieved, variations in muscle activity tend to become more consistent, reflecting a higher degree of coupling [45]. In other words, when two synergies operate in an In-phase pattern, the activation demand on individual muscles can be reduced. A previous study [42] found that enhanced coactivation of the triceps surae corresponded with reduced independent activation of the MG and improved gait stability. In this study, the AFF side more frequently exhibited a weighting pattern with increased MG contribution within Syn2, which likely serves to co-regulate ankle propulsion and postural control through a specific reorganization of this synergy’s internal distribution [46]. This shift in muscle weighting corresponds to the increased demand for foot stability in flatfoot gait. Previous biomechanical evidence indicates that individuals with flatfoot require increased plantarflexor output to stabilize the midfoot and facilitate push-off, thereby offsetting the mechanical disadvantage caused by the collapse of the medial longitudinal arch [7]. As the collapsed medial longitudinal arch reduces the foot’s natural ability to support body weight during push-off, a higher contribution from the MG likely helps maintain the necessary foot stiffness for effective propulsion [35].This internal reorganization toward agonist dominance within the synergy may mitigate the effects of unstable neural output by redistributing distal lower-limb activity to maintain stance-phase stability. Similar adaptive reorganizations of synergy patterns have also been reported in individuals with chronic ankle instability (CAI) [47]. Although such reorganized synergy structures may enhance postural stability in the stance phase, they may lead to reduced flexibility and increased energy consumption. From a kinetic chain perspective, the long-term reliance on synergy patterns featuring elevated MG weighting to maintain stability may alter the loading patterns of the proximal joints. The increased internal weighting of the MG, combined with abnormal foot alignment, potentially increases the loading of the medial compartment of the knee. This synergistic reorganization provides a possible neuromuscular rationale for the increased risk of medial knee osteoarthritis frequently observed in individuals with chronic flatfoot deformity [48]. Further research is needed to explore the differences in such synergistic reorganization under different tasks and loads, as well as to extend the investigation to other joints along the kinetic chain.

Walking is a complex movement that is coordinated and controlled by the central nervous system, involving multi-muscle synergistic activities [49, 50]. In traditional developed individuals, gait typically exhibits a high degree of rhythmicity and bilateral symmetry, which is crucial for maintaining motor efficiency and dynamic stability [50]. However, after impairment of the nervous system or musculoskeletal function, gait often exhibits varying degrees of asymmetry [51], which may lead to abnormal muscle activation. This study quantitatively analyzed the asymmetry of lower-limb muscle activity in UF individuals by combining NMF with VC to explore the imbalance and dominant deviation characteristics of bilateral muscle synergies during the stance phase. The combined method proposed in this study enables multidimensional analysis of muscle activity at the synergy level. It not only identifies the temporal expression of synergy modules and activation characteristics but also helps reveal the coupling patterns between synergy modules. While prior research [18] has explored information exchange between synergy modules, its conceptual framework and methodology were relatively complex and highly task-dependent. In contrast, the method employed in this study can more efficiently reveal the dynamic coordination characteristics of synergy modules. It features lower computational complexity, higher reproducibility, and broader applicability and particularly suitable for rhythmic movement tasks with periodic and repetitive characteristics in daily life and sports. The integration of NMF and VC reveals a temporal dimension of motor control beyond spatial weights. Given that basic synergy modules are largely preserved, VC dominance mapping illustrates how the nervous system strategically switches between functional synergies to maintain stability. This suggests that the reorganization of neural drive is manifested as shifts in coordination strategies rather than merely changes in activation intensity. In the future, this analytical method is expected to be further combined with electroencephalographic (EEG) recordings to explore the modular control strategies of the nervous system. It can also be extended to the field of rehabilitation to quantitatively evaluate the neural control remodeling process and the effectiveness of functional recovery before and after intervention. Rehabilitation could focus on strengthening intrinsic foot muscles to alleviate the compensatory demand on the medial gastrocnemius. Additionally, the quantitative features of synergy weighting and timing provide a framework for the design of personalized orthotic devices. By tracking these modular changes, clinicians can objectively monitor the progression of neuromuscular recovery during treatment. The results show that both sides had the same number of synergies and were primarily characterized by the In-phase pattern during the stance phase, while the AFF side exhibited a distinct internal reorganization of synergy weights. These results suggest that although functional imbalance leads to specific structural shifts within muscle synergies, the nervous system maintains overall gait stability by recalibrating the internal distribution of muscle contributions. The results provide a certain basis of understanding the neural control reorganization and muscle synergistic regulation mechanism in the gait of UF individuals, and also provide a certain theoretical reference for gait reconstruction and rehabilitation intervention based on muscle synergy.

This study has several limitations. Regarding the synergy control structure, the characterization was based on a parsimonious model determined by a strict reconstruction threshold during the stance phase, which may not account for subtle synergies used to fine-tune stability during the full gait cycle or more complex tasks. Furthermore, although our analysis confirmed a high degree of structural consistency in the extracted synergies, this functional homogeneity may not necessarily apply to individuals with more severe flatfoot deformities or those with concurrent neurological impairments. The current associations between synergy metrics and gait stability represent theoretical inferences that lack direct verification against clinical outcomes such as pain or injury risk. Future research should conduct clinical trials and longitudinal tracking to establish the functional relevance of these coordination patterns. The scope of this study was also limited to natural walking tasks. Future research should incorporate running or other high-intensity movement modes to expand the clinical applicability of these findings. Finally, as a cross-sectional study, the dynamic evolution of neural reorganization was not captured. Future longitudinal research should incorporate EEG and neuroimaging to fully elucidate the underlying control mechanisms.

## Conclusion

This study quantitatively analyzed the muscle synergy characteristics during the stance phase of gait in UF individuals using a combined NMF-VC approach. The study found that both lower limbs exhibited the same number of synergy modules and were primarily characterized by the In-phase pattern during the stance phase. The AFF side exhibited a distinct reorganization of internal synergy weights, suggesting that under functional imbalance, the nervous system may maintain gait stability by recalibrating the internal distribution of muscle contributions within specific synergies. The method proposed in this study is computationally simple and yields intuitive results, effectively revealing the dynamic coordination characteristics among synergy modules. It also provides a quantitative tool for EMG-based gait rehabilitation studies. Future work should include multi-task and longitudinal designs to explore changes in synergy patterns and the underlying neural control mechanisms.

## Data Availability

The datasets generated and/or analysed during the current study are not publicly available due ethical restrictions and the need to protect participant privacy, but are available from the corresponding author on reasonable request.

## Abbreviations

AFF: Affected
AI: Arch index
BF: Biceps femoris
CA: Coupling angle
CAV: Coupling angle variability
EMG: Electromyography
ES: Erector spinae
Gmax: Gluteus maximus
IDP-ROM: Inter-data-point range of motion
LG: Lateral gastrocnemius
MCA: Mean coupling angle
MG: Medial gastrocnemius
NMF: Non-negative matrix factorization
RF: Rectus femoris
sEMG: Surface electromyography
Syn: Synergy
TA: Tibialis anterior
UNAFF: Unaffected
UF: Unilateral flatfoot
VC: Vector coding
